# Internal Evaluation of a Machine Learning-Based Random Forest Model for Clinically Relevant Postoperative Pancreatic Fistula After Pancreaticoduodenectomy: Limited Incremental Value of Inflammatory–Nutritional Markers

**DOI:** 10.3390/jcm15218379

**Published:** 2026-10-28

**Authors:** Gizem Gunes, Adem Ozcan, Abdulkadir Unsal

**Affiliations:** Department of Surgical Oncology, Ankara City Hospital, 06800 Ankara, Türkiye

**Keywords:** pancreaticoduodenectomy, postoperative pancreatic fistula, random forest, machine learning, pancreatic duct diameter, neutrophil-to-lymphocyte ratio, monocyte-to-lymphocyte ratio

## Abstract

**Background and Objectives:** This study evaluated whether preoperative neutrophil-to-lymphocyte ratio (NLR) improves prediction of clinically relevant postoperative pancreatic fistula (CR-POPF) after pancreaticoduodenectomy. **Materials and Methods:** This single-center retrospective cohort included 120 of 158 patients operated on between January 2019 and March 2026; 38 lacked documented pancreatic duct diameter. CR-POPF followed the 2016 International Study Group on Pancreatic Surgery definition. A random forest incorporating duct diameter, estimated blood loss, and NLR was compared with a model omitting NLR and penalized logistic regression using 10-times-repeated stratified five-fold cross-validation. Post hoc clinicopathological sensitivity analyses added pancreatic texture and tumor origin, then tested inflammatory–nutritional markers individually. Discrimination, average precision, Brier score, calibration, and exploratory decision-curve analysis were assessed. **Results:** CR-POPF occurred in 16 patients (13.3%). Biochemical leak occurred in 28 (23.3%). The NLR-integrated model achieved an area under the receiver operating characteristic curve (AUC) of 0.721 (95% confidence interval [CI], 0.576–0.850), versus 0.760 for the comparator and 0.736 for penalized logistic regression. Expanded baseline AUC was 0.743; adding NLR yielded 0.715. Monocyte-to-lymphocyte ratio increased AUC numerically to 0.779, but the paired difference remained uncertain (ΔAUC, 0.037; 95% CI, −0.017 to 0.096). Other evaluated markers showed no clear incremental benefit. Postpancreatectomy acute pancreatitis occurred in seven (5.8%). **Conclusions:** NLR did not demonstrate incremental predictive value. The monocyte-to-lymphocyte ratio finding was hypothesis-generating. Small event numbers and predictor selection outside cross-validation limit interpretation; performance may remain optimistic. These findings do not support clinical implementation without independent external validation.

## 1. Introduction

Pancreaticoduodenectomy is the standard resection for many malignant diseases of the pancreatic head and periampullary region. Despite improvements in surgical technique and perioperative care, clinically relevant postoperative pancreatic fistula (CR-POPF) remains a major complication because it may lead to intra-abdominal infection, postpancreatectomy hemorrhage, invasive intervention, prolonged hospitalization, organ failure, or death. The 2016 update of the International Study Group on Pancreatic Surgery (ISGPS) separated biochemical leak from Grade B and Grade C fistulas and restricted the term POPF to clinically meaningful events [1].

Risk assessment has traditionally emphasized pancreatic texture, pancreatic duct diameter, pathology, estimated blood loss, body mass index, and related clinical features. These factors underpin the original Fistula Risk Score and later alternatives such as the Alternative and Updated Alternative Fistula Risk Scores [2,3]. External validations have shown that the contribution of individual variables and the discrimination of composite scores vary across operative approaches, reconstruction techniques, and populations, with several contemporary validations reporting only modest transportability [4,5,6,7].

Machine learning methods can represent nonlinear effects and interactions, but they do not eliminate the need for adequate sample size, careful internal evaluation, calibration assessment, and comparison with simpler models. Published machine learning approaches to POPF have reported moderate-to-high discrimination, although several relied on large datasets, imaging-derived features, or postoperative measurements that may not be universally available [8,9,10,11,12,13,14,15]. Inflammatory and nutritional markers are inexpensive and routinely measured, but their incremental predictive contribution beyond established anatomical and clinicopathological factors remains uncertain. Importantly, the timing of biomarker measurement differs substantially among studies: for example, postoperative day-3 NLR has been evaluated as a rule-out marker for POPF, whereas the present study focuses on preoperative inflammatory information [9,10,11,16,17].

The present study therefore evaluated an exploratory parsimonious random forest model combining pancreatic duct diameter, estimated blood loss, and NLR. The primary objective was not to claim a clinically deployable algorithm, but to quantify whether NLR added predictive information to an anatomical/intraoperative model under repeated internal evaluation. Predictor selection followed preliminary clinical and data exploration and was not preregistered; consequently, all performance estimates were interpreted as exploratory and potentially optimistic. A penalized logistic regression model was included as a simpler benchmark, and model assessment incorporated discrimination, average precision, Brier score, calibration, and exploratory decision-curve analysis.

## 2. Materials and Methods

### 2.1. Study Design and Cohort

This single-center retrospective cohort study used the records of patients who underwent pancreaticoduodenectomy in the Department of Surgical Oncology at Ankara City Hospital between January 2019 and March 2026. The retrospective source cohort comprised 158 patients. Pancreatic duct diameter was not documented in the operative records of 38 patients. Because pancreatic duct diameter was a required predictor in both random forest models and the penalized logistic regression model, these patients were excluded, leaving a final complete-case analytical cohort of 120 patients; no imputation was performed. Data were obtained from electronic medical records, operative reports, laboratory systems, radiology and intervention records, intensive care records, and discharge summaries. The study was approved by the Ankara City Hospital No. 1 Clinical Research Ethics Committee (TABED 1-26-2589; 20 May 2026). The ethics committee waived individual informed consent because the study was retrospective and used de-identified data.

To explore potential complete-case selection, available baseline and perioperative characteristics were additionally compared between the 120 patients included in the analytical cohort and the 38 patients excluded because pancreatic duct diameter was not documented. Continuous variables were compared using the Mann–Whitney U test and categorical variables using Fisher’s exact test, consistent with the primary descriptive analyses.

### 2.2. Operative Technique and Perioperative Management

All procedures were performed through an open surgical approach using a standard classic pancreaticoduodenectomy (Whipple procedure) with pyloric resection and gastrojejunostomy; pylorus-preserving Traverso–Longmire reconstruction was not used. Pancreatic reconstruction was performed as an end-to-side duct-to-mucosa pancreaticojejunostomy with a transanastomotic pancreatic duct stent. Invagination pancreaticojejunostomy and pancreaticogastrostomy were not used. A closed-suction drain was positioned adjacent to the pancreaticojejunostomy. Vascular resection was not performed in this cohort. Operations were performed by consultant surgical oncologists experienced in pancreatic surgery or under their direct supervision. The cohort comprised pancreatic ductal, ampullary, distal bile duct, and duodenal adenocarcinomas. Representative intraoperative views of this pancreaticojejunostomy technique are shown in Figure 1.

Drain-fluid amylase was routinely measured on postoperative days 1 and 3. Drain removal was not mandated on a single fixed postoperative day; it was guided by drain-amylase values, output volume and appearance, clinical examination, inflammatory findings, and imaging when clinically indicated. Nasogastric tubes were routinely removed on postoperative day 1. Patients were kept nil per os through postoperative day 3; a liquid diet was initiated on postoperative day 4 according to clinical tolerance, followed by oral nutritional supplementation. Routine tube enteral feeding was not used. Parenteral nutrition was used in patients who developed CR-POPF during periods in which oral intake was withheld. Immunonutrition was not administered routinely and was initiated selectively after consultation with the Clinical Nutrition Unit. The institutional perioperative antimicrobial regimen consisted of ceftriaxone plus metronidazole, initiated according to Infectious Diseases consultation and continued for 7 days. Somatostatin analogs were not administered routinely and were reserved for selected clinical indications.

These practices describe the institutional perioperative context rather than comparative evidence favoring a particular regimen. Their independent effects on CR-POPF were not estimated. Postoperative rescue treatments, including parenteral nutrition instituted after CR-POPF developed, were not used as prediction-model inputs.

### 2.3. Outcome Definition and Adjudication

The primary outcome was Grade B or Grade C CR-POPF according to the 2016 ISGPS definition [1]. POPF was defined as drain output of any measurable volume on or after postoperative day 3, with drain-fluid amylase activity greater than three times the upper limit of the institutional normal serum amylase level, accompanied by a clinically relevant condition directly attributable to the pancreatic fistula. Postoperative day 1 drain amylase was used for early postoperative surveillance but not as the diagnostic time point. An isolated amylase-rich drain output without a clinically relevant change in the postoperative course was classified as biochemical leak and was included in the non-CR-POPF group for binary modeling.

Grade B CR-POPF was defined by a fistula-related change in postoperative management without Grade C features, including prolonged drainage for more than three weeks, percutaneous or endoscopic drainage or repositioning, or other fistula-directed nonoperative treatment. Grade C CR-POPF was defined by fistula-related reoperation, single or multiple organ failure, or death. Organ failure was considered present when fistula-related respiratory, renal, or circulatory failure required reintubation, hemodialysis, or inotropic support for more than 24 h, respectively [1].

Outcome status was retrospectively verified by the study investigator, a surgical oncologist, using laboratory results, daily clinical notes, drain records, imaging, interventional procedure reports, reoperation records, intensive care documentation, and discharge summaries. The source analytical database stored Grade B and Grade C events as a combined CR-POPF category; therefore, separate Grade B and Grade C counts could not be reconstructed reliably. A formal independent two-reviewer adjudication process was not performed, and outcome adjudication was not blinded to predictor information. Postpancreatectomy acute pancreatitis (PPAP) was additionally assessed according to the ISGPS definition: an acute inflammatory condition of the pancreatic remnant beginning within the first 3 postoperative days, requiring sustained postoperative hyperamylasemia above the institutional upper limit of normal for at least the first 48 h, together with clinically relevant features and radiological findings compatible with PPAP [18]. Fulfilment of the ISGPS diagnostic criteria for PPAP was retrospectively confirmed in all seven cases using serum amylase measurements, clinical documentation, and radiological findings from the medical records. PPAP was treated as a postoperative outcome and was not entered as a model predictor to avoid temporal information leakage.

### 2.4. Candidate Predictors and Derived Indices

Candidate and descriptive variables were age, sex, body mass index (BMI), diabetes mellitus, preoperative hemoglobin, total bilirubin, albumin, C-reactive protein (CRP), lymphocyte count, platelet count, neutrophil count, monocyte count, pancreatic texture, pancreatic duct diameter, operative time, estimated blood loss, and tumor origin/pathological diagnosis. Laboratory indices were recalculated from the last available preoperative raw measurements recorded in the dataset. During revision, BMI, monocyte count, and detailed tumor origin were retrieved from the source records. Pancreatic texture was documented intraoperatively by the operating surgical team and coded as 1 = soft and 2 = firm/hard. Tumor origin was categorized as pancreatic ductal adenocarcinoma, ampullary adenocarcinoma, distal cholangiocarcinoma, or duodenal adenocarcinoma. Pathological diagnosis, primary anatomic site, and indication for pancreaticoduodenectomy were concordant in this cohort; therefore, a single four-level tumor-origin variable was used in sensitivity modeling to avoid redundant representation of the same information. Data-quality checks included allowed-value and range review, exact-duplicate-row assessment, and recalculation of derived indices from their component variables; no exact duplicate patient rows were identified.

The four-level tumor-origin variable is a clinicopathological classification. Concordance between pathological diagnosis, anatomical site, and surgical indication does not by itself establish that the same diagnostic label was known before or during surgery. Accordingly, analyses incorporating tumor origin are reported as retrospective clinicopathological sensitivity analyses, not as validation of an intraoperative clinical tool. Availability and coding of this information at a prespecified prediction time must be established before prospective application.

- NLR = neutrophil count/lymphocyte count.
- MLR = monocyte count/lymphocyte count.
- PNI = albumin (g/L) + 5 × lymphocyte count (×10^9^/L).
- PLR = platelet count/lymphocyte count.
- CALLY = albumin (g/L) × lymphocyte count (×10^9^/L)/[CRP (mg/L) × 10], which is unit-equivalent to the original formulation using albumin in g/dL, lymphocytes per µL, and CRP in mg/dL [19].

Prior research on NLR after pancreatic surgery provided the rationale for focusing on this marker. Garnier et al. evaluated postoperative day-3 NLR for excluding POPF after pancreaticoduodenectomy [17]. This motivated the distinct hypothesis that the same ratio, measured before surgery, might add predictive information to pancreatic duct diameter and estimated blood loss. The postoperative evidence was not treated as proof of preoperative predictive value or superiority over other inflammatory–nutritional indices. NLR was therefore the marker under investigation, rather than an established best-performing marker. With only 16 CR-POPF events, the original exploratory model was limited to duct diameter, blood loss, and NLR, rather than a multibiomarker panel.

This rationale should be distinguished from statistical evidence favoring NLR. PLR is also derived from routine blood counts, while PNI and CALLY incorporate albumin and, for CALLY, CRP. These differences in composition do not themselves establish a predictive advantage for NLR. NLR was not selected because of a statistically significant univariable association; likewise, non-significant univariable comparisons for PLR, PNI, and CALLY do not show that those markers lack incremental predictive information. Monocyte counts were retrieved during revision, and MLR was evaluated in the subsequent sensitivity analysis rather than in the original three-predictor model.

The initial selection nevertheless followed preliminary clinical and data exploration, was not preregistered, and was performed outside the cross-validation loop. The clinical and literature-based rationale above is not a fully reproducible account of how individual exploratory findings influenced that choice, and the choice cannot be assumed to have been independent of outcome-informed exploration. In particular, the analysis does not establish NLR as the winner of a prospectively specified comparison based on p values, AUC thresholds, or feature-importance rankings. Cross-validation therefore evaluated prediction conditional on the chosen predictors, not the process by which NLR had been selected. This distinction limits interpretation of internal performance estimates and is not resolved by the absence of a significant univariable association or by holding model hyperparameters constant.

A two-variable random forest comparator contained only pancreatic duct diameter and estimated blood loss. To address concerns regarding omission of established risk factors and alternative inflammatory–nutritional markers, a reviewer-requested post hoc sensitivity analysis was added. The expanded baseline random forest included pancreatic duct diameter, estimated blood loss, pancreatic texture, and tumor origin; tumor origin was represented by three indicator variables with pancreatic ductal adenocarcinoma as the reference category. NLR, MLR, PLR, PNI, and CALLY were then added separately, one at a time, to the same expanded baseline model. The markers were not entered simultaneously because of their biological overlap and the limited number of CR-POPF events. These expanded analyses were explicitly considered exploratory and were not used to select a deployable clinical model. This reviewer-requested comparison is separate from the initial marker choice and does not retrospectively validate the selection of NLR.

### 2.5. Model Development and Internal Evaluation

Random forest models were implemented in Python with scikit-learn version 1.8.0. For the primary and expanded sensitivity analyses, one fixed hyperparameter configuration was used in every resampling iteration; no within-study hyperparameter search was performed. The configuration comprised 500 trees, a maximum depth of 3, a minimum leaf size of 2, and square-root feature sampling. Class weighting was not applied. Class imbalance was addressed during model evaluation by reporting average precision, calibration, and Brier score in addition to the AUC. A penalized logistic regression model with L2 regularization, standardized predictors, and the same three primary predictors was fitted as a simpler benchmark for the primary analysis. Model outputs were continuous predicted probabilities; no fixed clinical classification threshold was proposed. The reviewer-requested marker comparisons used the same random-forest configuration and the same resampling scheme for every expanded model so that differences reflected predictor content rather than model tuning.

Internal evaluation used 10-times-repeated stratified five-fold cross-validation. Each patient therefore received 10 out-of-fold predicted probabilities, which were averaged to obtain one patient-level estimate for each model. Discrimination was quantified using the area under the receiver operating characteristic curve (AUC), and performance under class imbalance was summarized with average precision. Overall prediction error was assessed with the Brier score. The null-model Brier score was calculated from the event prevalence, and the Brier skill score was defined as 1 − (model Brier score/null-model Brier score). All model development and internal performance evaluation used the same single-center source cohort throughout out-of-fold resampling; no separate external evaluation dataset was available. The analysis required complete values for all model predictors, and no imputation or implementation strategy for missing inputs was developed.

Calibration was summarized by calibration intercept and slope using the out-of-fold predictions and displayed with five quantile-based groups. Exploratory decision-curve analysis evaluated net benefit over threshold probabilities from 0.05 to 0.40 [20]. Confidence intervals for AUC, average precision, Brier score, and paired differences in AUC were derived from 3000 patient-level bootstrap resamples. Calibration estimates were reported without confidence intervals and were interpreted cautiously because only 16 events were available. Because the event count was small, all model-evaluation and clinical-utility analyses were considered exploratory.

### 2.6. Statistical Analysis

In the revised analysis, normality of continuous variables was formally assessed using the Shapiro–Wilk test. Several clinically important variables, including hemoglobin, bilirubin, CRP, platelet count, neutrophil count, BMI, NLR, MLR, PLR, CALLY, pancreatic duct diameter, operative time, and estimated blood loss, showed statistically significant departures from normality. Given these mixed distributions and the small CR-POPF subgroup, continuous variables are presented as median and interquartile range and were compared using the Mann–Whitney U test. Binary categorical variables are presented as number and percentage and were compared using Fisher’s exact test. The four-category tumor-origin distribution was compared using the Fisher–Freeman–Halton exact test. Univariable discrimination of pancreatic duct diameter was quantified using the AUC, with smaller duct diameter coded as higher predicted risk; its 95% confidence interval was obtained from 3000 bootstrap resamples. A univariable logistic regression model estimated the odds ratio for CR-POPF per 1 mm increase in pancreatic duct diameter. All tests were two-sided, and *p* < 0.05 was considered statistically significant. No formal sample-size calculation was performed because all available eligible complete cases in the retrospective source cohort were used. The limited number of events restricted model complexity and precluded reliable subgroup-performance analyses. Reporting followed TRIPOD + AI principles for an exploratory prediction-model study [21].

### 2.7. Open Science, Registration, and Patient and Public Involvement

The study and analytical plan were not prospectively registered or preregistered. Model configuration, predictor handling, and performance assessment are described in Section 2.4, Section 2.5 and Section 2.6. De-identified patient-level data are subject to ethical and institutional restrictions. Patients and members of the public were not involved in the design, conduct, interpretation, reporting, or dissemination planning of this retrospective study.

## 3. Results

### 3.1. Outcome Distribution and Baseline Characteristics

The retrospective source cohort included 158 patients; 38 were excluded because pancreatic duct diameter was not documented in the operative records, leaving 120 patients for analysis (Figure 2). Among the 120 patients, 76 (63.3%) had no pancreatic leak, 28 (23.3%) had biochemical leak, and 16 (13.3%) had CR-POPF. Because Grade B and Grade C were stored as one combined category in the source dataset, their separate frequencies could not be reported. The median age was 69.0 years, 79 patients (65.8%) were male, 67 (55.8%) had diabetes mellitus, and the median BMI was 26.85 kg/m^2^. The pathological indications for pancreaticoduodenectomy were pancreatic ductal adenocarcinoma in 68 patients (56.7%), ampullary adenocarcinoma in 28 (23.3%), distal cholangiocarcinoma in 18 (15.0%), and duodenal adenocarcinoma in 6 (5.0%). No pancreatic neuroendocrine neoplasms or pancreatic cystic neoplasms were represented in the analytical cohort.

To assess the potential for systematic complete-case selection, the 120 patients included in the analytical cohort were compared with the 38 patients excluded because pancreatic duct diameter was not documented. No statistically significant differences were observed in the available demographic, laboratory, inflammatory–nutritional, pancreatic texture, or perioperative characteristics between the included and excluded groups (all *p* > 0.05; Supplementary Table S1). The absence of statistically significant differences does not demonstrate equivalence or establish that duct-diameter documentation was missing at random. Selection bias related to unmeasured factors or to the missing-data mechanism cannot be excluded.

Pancreatic duct diameter was significantly smaller in patients with CR-POPF than in patients without CR-POPF (median, 2.0 mm [IQR, 1.0–2.0] vs. 3.0 mm [IQR, 2.0–3.25]; *p* < 0.001). On univariable analysis, smaller pancreatic duct diameter discriminated CR-POPF with an AUC of 0.753 (95% CI, 0.625–0.860). In univariable logistic regression, each 1 mm increase in pancreatic duct diameter was associated with lower odds of CR-POPF (OR, 0.35; 95% CI, 0.18–0.69; *p* = 0.003). Diabetes mellitus was more frequent in the CR-POPF group (75.0% vs. 52.9%), and estimated blood loss was numerically higher (500 vs. 300 mL), but neither difference reached statistical significance. Soft pancreatic texture occurred in 75.0% of both groups (*p* = 1.000). The overall distribution of tumor origin differed between groups (Fisher–Freeman–Halton *p* = 0.025), although the sparse category-specific event counts precluded stable disease-specific inference. BMI, monocyte count, NLR, MLR, PNI, PLR, CALLY, albumin, and CRP did not differ significantly between groups (Table 1). PPAP occurred in 7 of 120 patients (5.8%): 6 of 104 patients without CR-POPF (5.8%) and 1 of 16 patients with CR-POPF (6.3%; Fisher’s exact *p* = 1.000).

### 3.2. Cross-Validated Model Performance

The integrated random forest model achieved an AUC of 0.721 (95% CI, 0.576–0.850), average precision of 0.294 (95% CI, 0.162–0.542), and Brier score of 0.108 (95% CI, 0.069–0.151). The null-model Brier score was 0.116, corresponding to a modest Brier skill score of 0.069.

The anatomical/intraoperative random forest comparator achieved an AUC of 0.760 (95% CI, 0.638–0.868), average precision of 0.311 (95% CI, 0.176–0.559), and Brier score of 0.103 (95% CI, 0.066–0.144). Adding NLR reduced rather than improved discrimination numerically (ΔAUC, −0.039; 95% CI, −0.096 to 0.019). Penalized logistic regression using duct diameter, blood loss, and NLR achieved an AUC of 0.736 (95% CI, 0.596–0.856), average precision of 0.338 (95% CI, 0.179–0.593), and Brier score of 0.107 (95% CI, 0.072–0.146). In the paired comparison of models using the same three predictors, the AUC difference between the integrated random forest and penalized logistic regression was −0.014 (95% CI, −0.075 to 0.046), indicating no clear discrimination advantage for random forest (Table 2 and Table 3; Figure 3).

Given the limited number of CR-POPF events (*n* = 16) and the fact that predictor selection was performed after preliminary data exploration outside the cross-validation loops, these performance estimates should be interpreted as exploratory and may remain optimistic. The reported bootstrap confidence intervals quantify sampling uncertainty conditional on the selected predictor set, but they do not account for additional optimism introduced by data-driven predictor selection.

Predictions are patient-level averages of out-of-fold probabilities from 10-times-repeated stratified five-fold cross-validation. Confidence intervals for AUC, average precision, Brier score, and paired AUC differences were obtained from 3000 patient-level bootstrap resamples. The null-model Brier score was 0.116. Calibration estimates were based on cross-validated predictions and are imprecise because only 16 events were available.

### 3.3. Expanded Clinicopathological and Inflammatory–Nutritional Sensitivity Analyses

In the reviewer-requested retrospective clinicopathological sensitivity analysis, adding pancreatic texture and tumor origin to pancreatic duct diameter and estimated blood loss did not improve discrimination compared with the original two-variable comparator (expanded baseline AUC, 0.743 [95% CI, 0.601–0.862] vs. original comparator AUC, 0.760; paired ΔAUC, −0.017 [95% CI, −0.086 to 0.046]). Adding preoperative NLR to the expanded baseline further reduced discrimination (AUC, 0.715 [95% CI, 0.569–0.836]; ΔAUC, −0.028 [95% CI, −0.055 to −0.003]). Among the alternative markers evaluated one at a time, MLR produced a numerical increase in AUC to 0.779 (95% CI, 0.650–0.888), with higher average precision (0.419 vs. 0.310) and a slightly lower Brier score (0.102 vs. 0.106); however, the paired ΔAUC confidence interval included zero (ΔAUC, 0.037; 95% CI, −0.017 to 0.096). PLR, PNI, and CALLY did not demonstrate clear incremental benefit. Because these analyses were post hoc, involved only 16 events, and examined several candidate markers, the MLR finding was considered hypothesis-generating rather than confirmatory (Table 4).

### 3.4. Calibration and Exploratory Clinical Utility

For the integrated random forest, the calibration intercept was −0.092 and the calibration slope was 0.971. Although these values may appear favorable, they should be interpreted with particular caution because only 16 CR-POPF events were available and predictor selection was performed outside the cross-validation loops. The random forest comparator had an intercept of −0.185 and slope of 0.900, while penalized logistic regression had an intercept of −0.059 and slope of 1.093 (Table 2). Grouped calibration curves showed substantial sampling variability, particularly in the higher predicted-risk groups (Figure 4), and the apparent calibration performance may therefore be optimistic.

Exploratory decision-curve analysis suggested small positive net benefit for all three models across portions of the examined threshold range, but the integrated model did not demonstrate a consistent advantage over the anatomical/intraoperative comparator or penalized logistic regression (Figure 5). These findings do not establish clinical utility.

## 4. Discussion

This study evaluated whether a routinely available preoperative inflammatory marker improved CR-POPF prediction beyond established anatomical and intraoperative information. The primary analysis did not demonstrate incremental predictive value from adding NLR to pancreatic duct diameter and estimated blood loss. Adding NLR did not improve discrimination or overall prediction error and did not demonstrate a consistent decision-curve advantage. Calibration point estimates were numerically more favorable than those of the two-variable comparator, but were imprecise and potentially optimistic given the small event count. A reviewer-requested retrospective clinicopathological analysis then incorporated pancreatic texture and tumor origin and tested several inflammatory–nutritional markers individually. The expanded baseline itself did not outperform the original two-variable comparator, and adding NLR again reduced discrimination. MLR yielded a numerical increase in AUC and average precision, but the paired ΔAUC confidence interval crossed zero. In the setting of only 16 CR-POPF events and multiple exploratory marker comparisons, this isolated signal should be viewed as hypothesis-generating rather than evidence of a validated incremental predictor.

Pancreatic duct diameter showed moderate univariable discrimination (AUC, 0.753), consistent with the biological and technical basis of established fistula scores [2,3,4]. Each 1 mm increase in duct diameter was associated with lower odds of CR-POPF in univariable logistic regression. A narrow duct increases the difficulty of duct-to-mucosa reconstruction and is repeatedly associated with CR-POPF. Estimated blood loss also contributed to the comparator model, although its reproducibility and independent predictive value have varied across external validations.

Pancreatic texture and pathology/tumor origin are established components or correlates of contemporary Fistula Risk Scores, and BMI is included in several alternative approaches [2,3,5,6]. In the present cohort, soft pancreatic texture was observed in 75.0% of both outcome groups, and adding texture together with tumor origin did not improve cross-validated discrimination. This cohort-specific finding is not evidence that these established clinical factors are unimportant. No PNETs or PCNs were represented, and the four adenocarcinoma groups contained sparse and imbalanced event counts, limiting histology-specific inference. External score performance must also be considered in context: Cheok et al. reported AUCs of 0.662–0.675 for four Fistula Risk Score variants in 724 patients, reinforcing the importance of population-specific validation and calibration [7].

The homogeneous operative approach in this cohort—classic Whipple resection, duct-to-mucosa pancreaticojejunostomy, transanastomotic stenting, no vascular resection, and standardized drain-amylase measurement—reduces some procedural heterogeneity but also limits generalizability to other reconstruction and perioperative strategies.

Inflammatory and nutritional biomarkers are attractive because they are inexpensive and widely available, but published findings are heterogeneous and often address different clinical time points. Preoperative CRP-to-albumin ratio has been associated with POPF after pancreaticoduodenectomy [10], and preoperative PNI has been associated with progression from biochemical leak to CR-POPF [16]. In contrast, Garnier et al. evaluated NLR on postoperative day 3 and found its value primarily as a rule-out marker for POPF [17], a substantially different setting from the present study’s preoperative incremental prediction question. In our data, preoperative NLR did not add value to either the primary or expanded baseline. MLR was the only additional marker associated with a numerical increase in AUC, although the paired confidence interval included zero. PLR, PNI, and CALLY did not provide clear incremental discrimination. These findings argue against selecting inflammatory markers solely on the basis of apparent performance in a small development cohort and support independent validation before any marker is incorporated into perioperative decision-making.

An association with an outcome in a selected population is also distinct from incremental predictive value in all patients undergoing PD. For example, the PNI study by Aydin et al. evaluated progression among patients who had already developed a biochemical leak [16], whereas the present analysis evaluated CR-POPF across the complete analytical cohort. Differences in the target population and prediction time therefore limit direct comparison of marker performance.

The revised analysis also highlights why model evaluation should extend beyond AUC. The primary integrated model’s Brier score was only modestly better than the prevalence-only null model, calibration estimates were uncertain, and random forest did not clearly outperform penalized logistic regression. The expanded analyses produced the same broader message: additional biological and clinicopathological variables did not automatically translate into more stable prediction. Calibration, precision–recall performance, paired uncertainty estimates, and independent validation are therefore central to interpretation.

Recent machine learning studies provide useful methodological contrasts rather than direct performance benchmarks. Verma et al. developed an XGBoost model using 12,281 national-registry patients and evaluated it in a separate 445-patient institutional cohort, reporting an external-validation AUC of 0.79 [13]. Lee et al. combined clinical, body-composition, and CT information in a 1333-patient development/temporal-test framework; their selected preoperative model achieved an AUC of 0.68 for CR-POPF in the test set [14]. Wang et al. studied 210 laparoscopic PD patients with 34 CR-POPF events and reported a held-out random-forest AUC of 0.747 [15]. These differences in sample size, operative approach, inputs, and validation design preclude concluding that one algorithm is superior based on cross-study AUC comparisons. In particular, repeated internal cross-validation in the present 120-patient cohort is not a substitute for the independent validation used in larger studies.

PPAP occurred in 5.8% of this cohort and was reported as a postoperative outcome. It was deliberately excluded from prediction modeling because using a postoperative complication as if it were available preoperatively or intraoperatively would introduce temporal information leakage [18].

This study has several limitations. First, it was retrospective and single-center. Thirty-eight of 158 potentially eligible patients (24.1%) were excluded because pancreatic duct diameter, a required model predictor, was not documented. Although included and excluded patients did not differ significantly across the available measured characteristics, residual complete-case selection bias remains possible. Second, only 16 CR-POPF events were available. This small event count results in wide uncertainty, limits effective model dimensionality, and increases the risk that cross-validated estimates remain unstable. With five-fold stratification, each validation fold contains only approximately three or four events; repeating the folds does not create additional independent outcome information. Small changes in the patients allocated to a fold can therefore alter discrimination and calibration estimates. Repeated cross-validation reduces dependence on a single train/test split but cannot eliminate optimism arising from small-sample development or data-informed predictor selection. Third, primary predictor selection occurred after preliminary exploration and outside the resampling loops. The clinical and literature-based rationale for choosing NLR does not reconstruct the full sequence of the initial exploration; independence of marker selection from the observed outcomes therefore cannot be assumed. The reported bootstrap confidence intervals reflect sampling uncertainty conditional on the selected predictor set but do not fully account for selection-induced optimism. Fourth, the expanded analyses incorporating pancreatic texture, tumor origin, and multiple inflammatory–nutritional markers were post hoc responses to peer review. They should be interpreted strictly as sensitivity analyses; the four pathology groups were imbalanced and contained sparse event counts, including no CR-POPF events among distal cholangiocarcinoma cases. The clinicopathological tumor-origin label also does not establish that identical information was available at the intended prediction time; its inclusion is therefore interpreted as a retrospective sensitivity analysis rather than as evidence of intraoperative deployability. Fifth, Grade B and Grade C events were combined in the source analytical database, preventing reliable separate reporting, and outcome adjudication was performed by one investigator without independent duplicate review. Sixth, surgeon-specific volume was unavailable and the drain-removal protocol was clinically individualized rather than fixed. Finally, no independent external validation cohort was available, and all calibration and decision-curve findings should be considered exploratory.

Before any clinical tool could be derived, the model and predictor definitions would need to be frozen and tested without refitting in an independent multicenter cohort. A collaboration should first agree on a common protocol, appropriate ethics and data-sharing arrangements, a prespecified prediction time, and a shared data dictionary. Participating centers would need harmonized 2016 ISGPS outcome definitions, separate recording of Grade B and Grade C events, standardized duct-diameter and texture assessment, common laboratory time points, and tumor-origin coding based only on information available at the prediction time. A validation sample-size calculation should be based on the desired precision of the performance and calibration estimates, rather than on the size of the present development cohort.

The frozen model should then be applied to consecutively assembled independent patients using a prespecified approach to missing inputs, with discrimination, average precision, Brier score, calibration intercept and slope, and decision-curve analysis reported. Case mix, reconstruction technique, and perioperative pathways should be documented to assess transportability across centers. Only after the unmodified model has been evaluated should recalibration or model updating be considered; any updated model would require further evaluation. These steps describe a proposed validation framework, not a collaboration or external validation already completed in this study.

Despite these limitations, the analysis has strengths: the endpoint was aligned with the 2016 ISGPS framework, operative and drain-monitoring practices were described explicitly, prediction estimates were generated out of fold under repeated stratified cross-validation, a simpler penalized-regression benchmark was included, and calibration, Brier score, average precision, and decision-curve analysis were reported rather than relying on AUC alone.

## 5. Conclusions

In this single-center cohort of 120 patients undergoing pancreaticoduodenectomy, pancreatic duct diameter showed moderate univariable discrimination for CR-POPF. The primary two-variable anatomical/intraoperative comparator performed numerically better than the NLR-integrated model, and random forest did not clearly outperform penalized logistic regression. The reviewer-requested retrospective clinicopathological expansion with pancreatic texture and tumor origin did not improve baseline discrimination, and NLR again showed no incremental value. Among alternative inflammatory–nutritional markers, MLR showed a numerical but statistically uncertain improvement that should be considered hypothesis-generating. These findings do not support clinical implementation of the present models or biomarkers. Larger multicenter cohorts with separate Grade B and Grade C adjudication, prespecified predictor handling, and true external validation are required before clinical use can be considered.

## Figures and Tables

**Figure 1:**
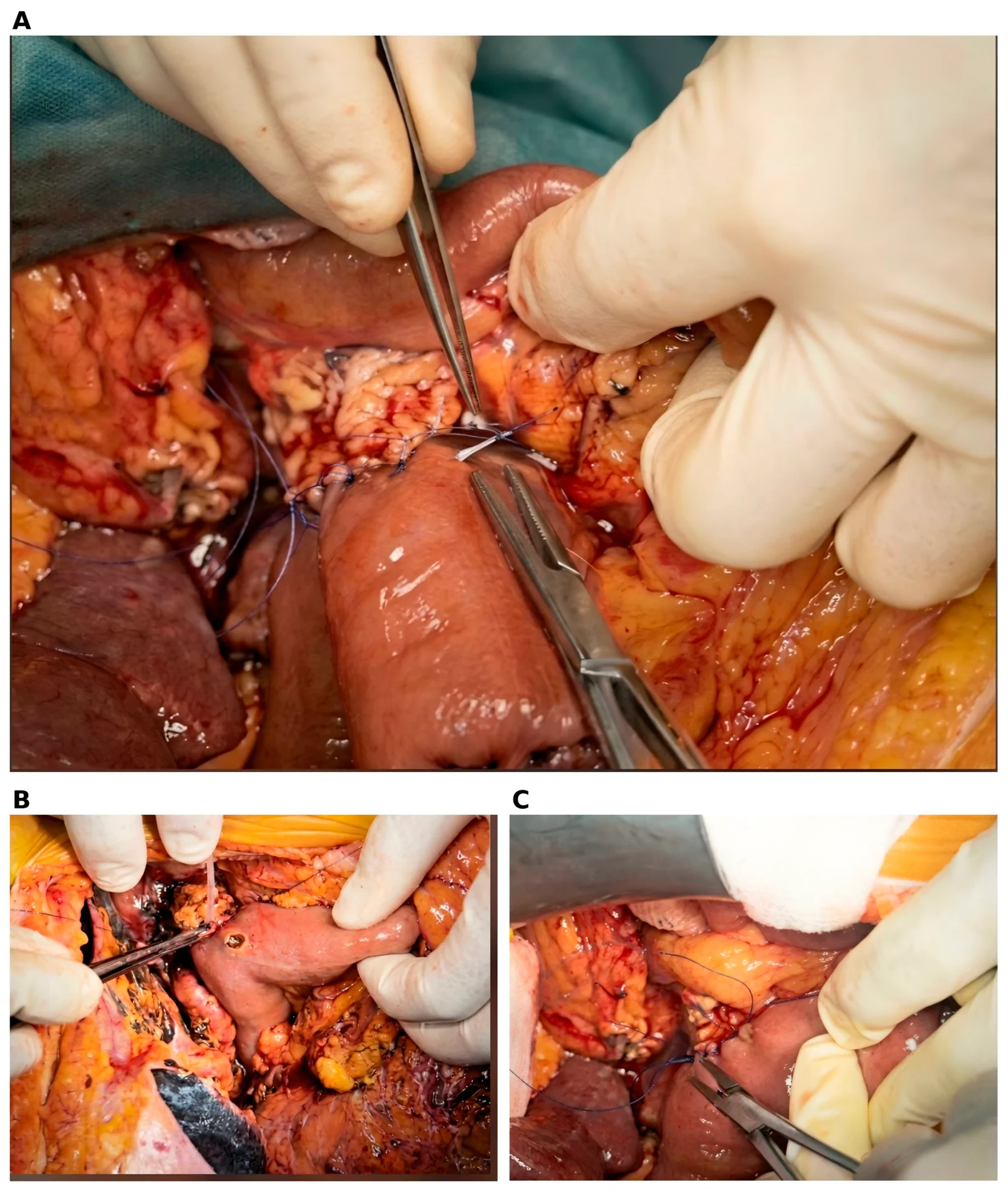
Representative intraoperative views of the end-to-side duct-to-mucosa pancreaticojejunostomy technique described in Section 2.2. (**A**) Pancreatic remnant and jejunal limb with sutures visible at the anastomotic interface. (**B**) Jejunal opening and pancreatic duct stent. (**C**) Approximation of the pancreatic remnant and jejunal limb with sutures in place.

**Figure 2:**
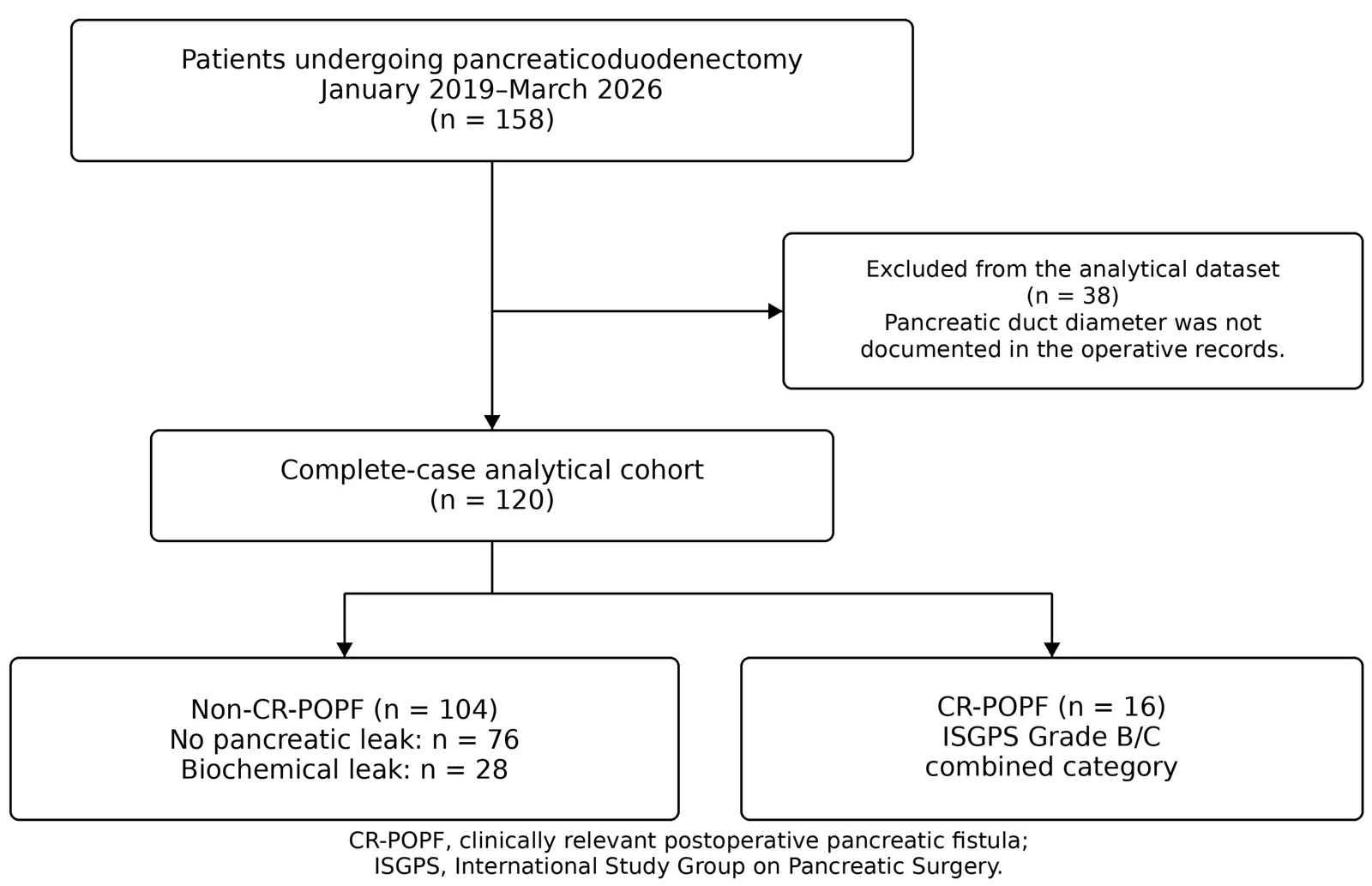
Study flow diagram. The retrospective source cohort comprised 158 patients; 38 were excluded because pancreatic duct diameter was not documented in the operative records, leaving 120 patients. CR-POPF, clinically relevant postoperative pancreatic fistula; ISGPS, International Study Group on Pancreatic Surgery.

**Figure 3:**
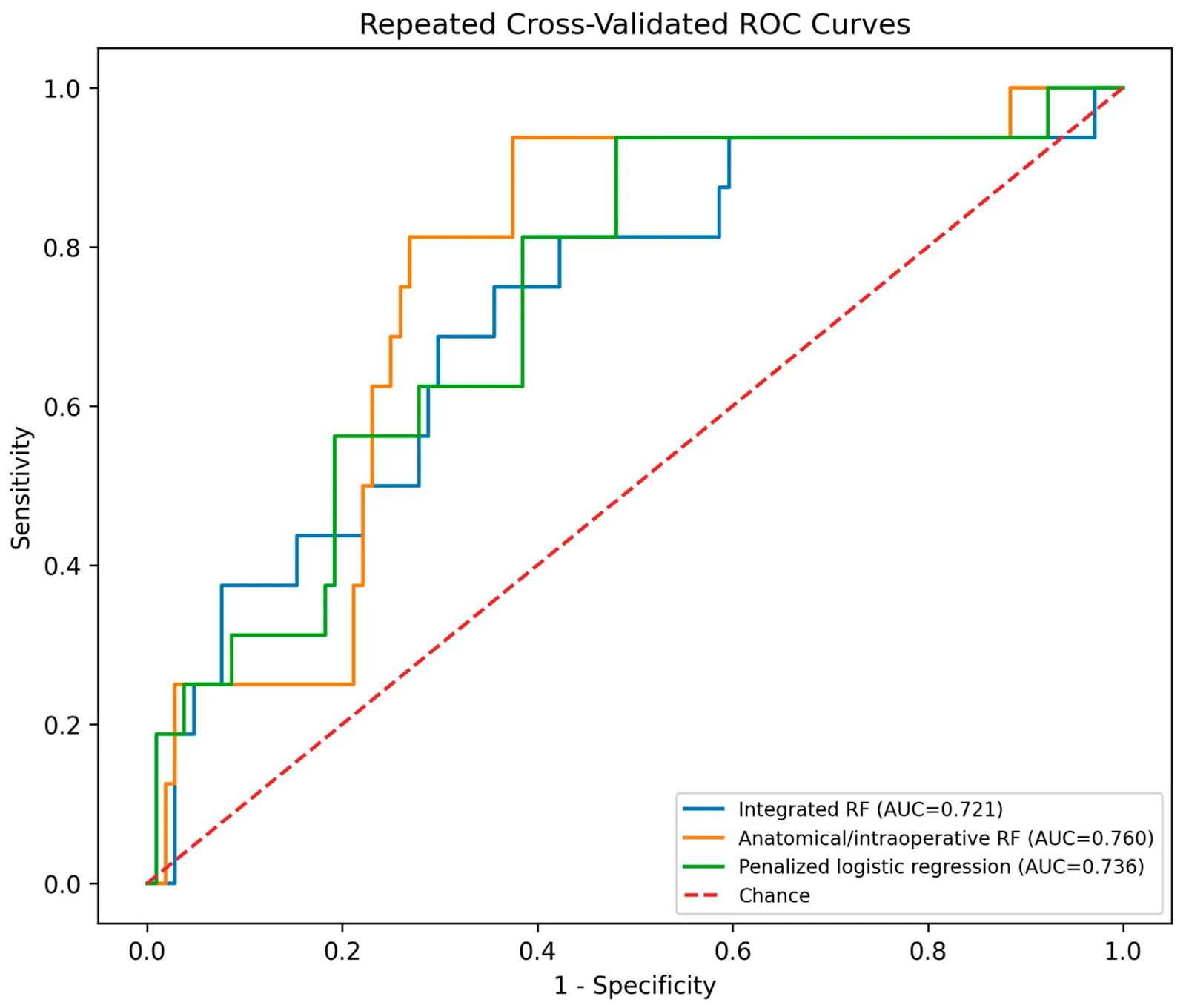
Repeated cross-validated receiver operating characteristic curves for the integrated random forest, anatomical/intraoperative random forest comparator, and penalized logistic regression model.

**Figure 4:**
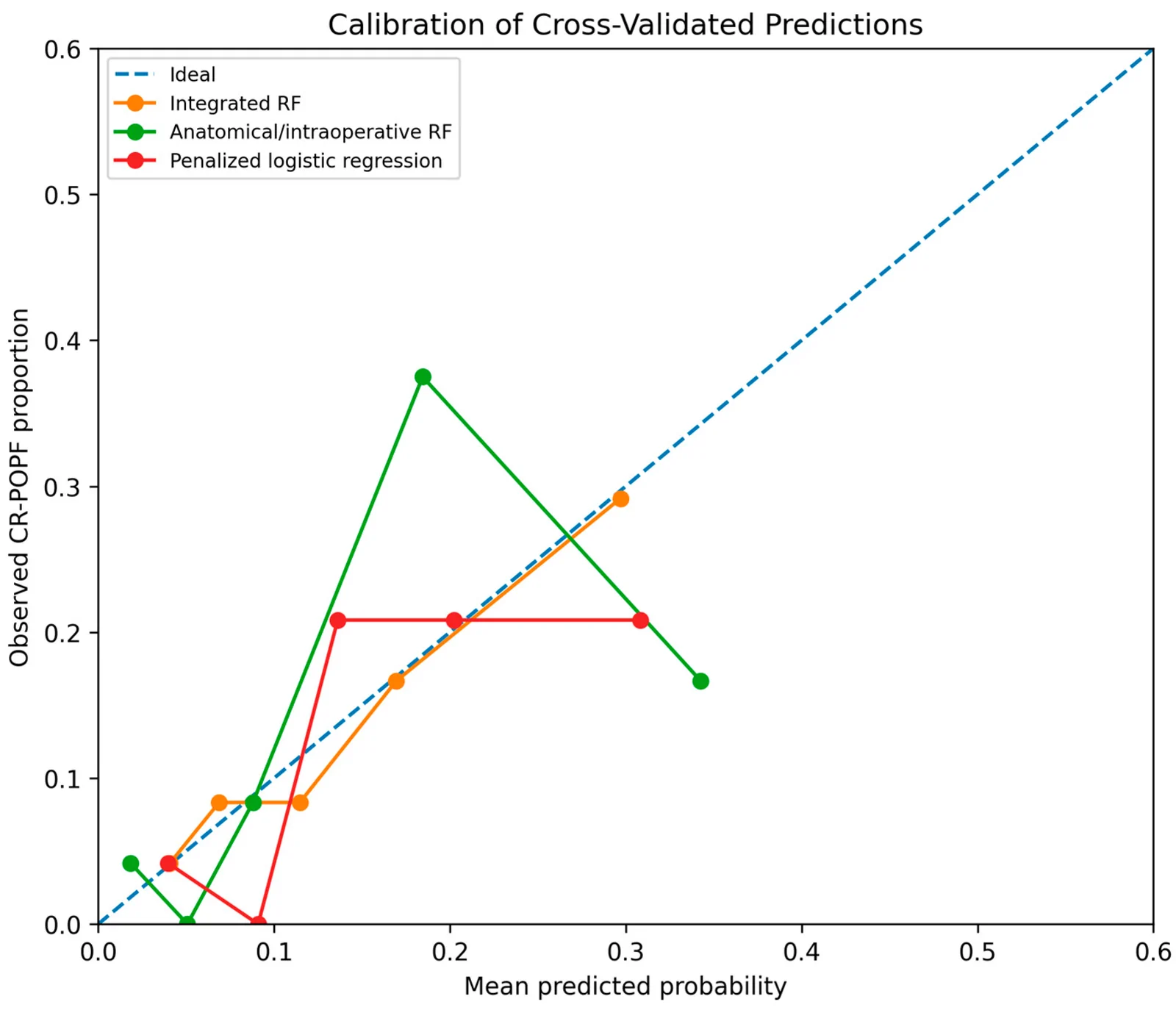
Calibration of patient-level cross-validated predictions. Points represent five quantile-based groups. The apparent irregularity reflects the small number of CR-POPF events and should be interpreted cautiously.

**Figure 5:**
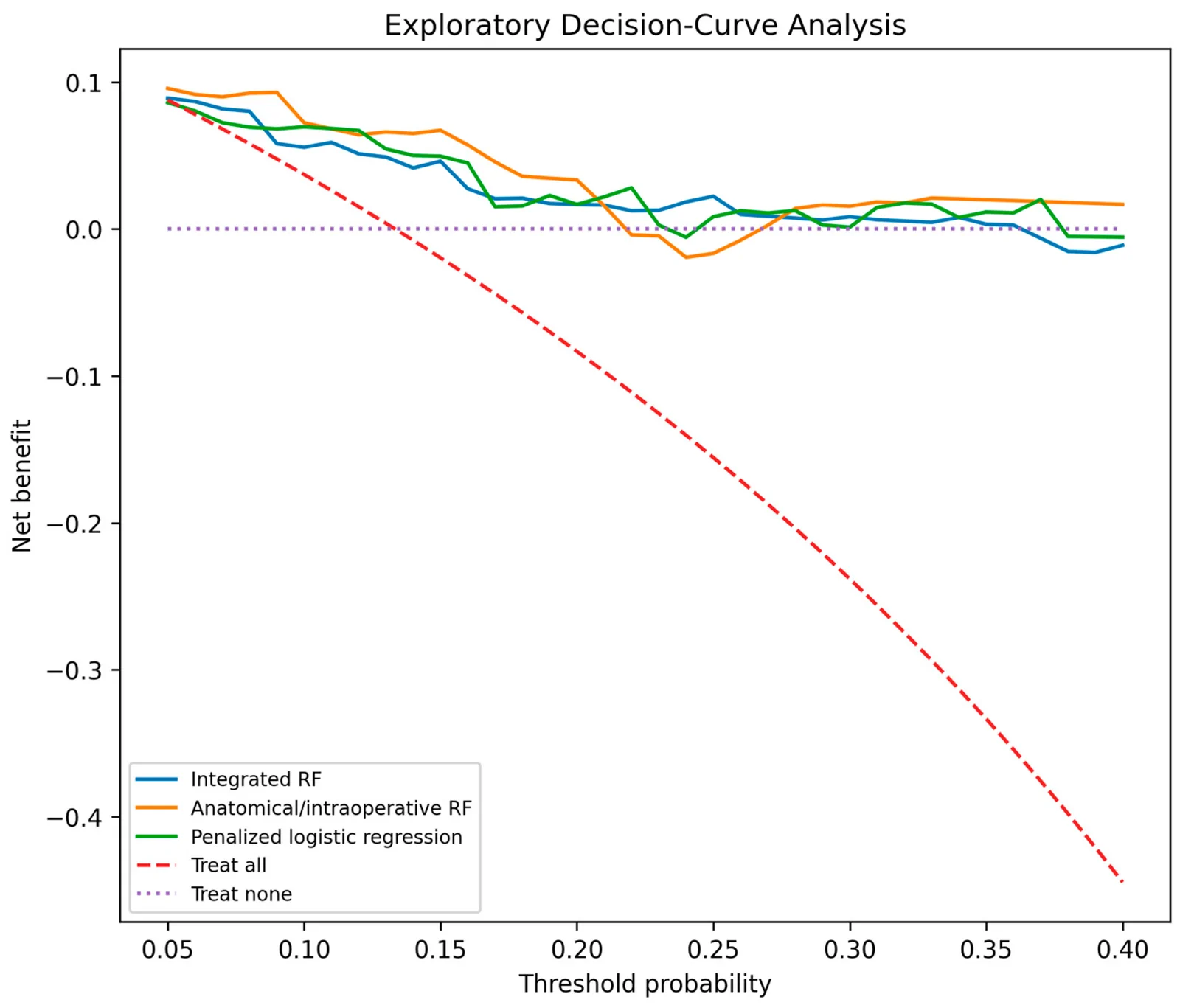
Exploratory decision-curve analysis across threshold probabilities of 0.05–0.40. The integrated model did not show a consistent net-benefit advantage over the comparator or penalized logistic regression.

**Table 1:** Baseline, clinicopathological, and perioperative characteristics according to CR-POPF status.

Variable	Overall (*n* = 120)	Non-CR-POPF (*n* = 104)	CR-POPF (*n* = 16)	*p* Value
Age, years	69.0 (63.8–76.0)	69.0 (63.0–76.0)	69.0 (64.8–73.8)	0.847
BMI, kg/m^2^	26.85 (24.15–30.00)	26.85 (23.98–30.00)	26.90 (24.73–28.23)	0.714
Male sex	79 (65.8)	68 (65.4)	11 (68.8)	1.000
Diabetes mellitus	67 (55.8)	55 (52.9)	12 (75.0)	0.112
Hemoglobin, g/dL	13.4 (11.7–14.7)	13.4 (11.8–14.7)	13.6 (11.5–14.4)	0.892
Total bilirubin, mg/dL	0.98 (0.61–3.09)	0.95 (0.59–3.00)	1.27 (0.82–4.52)	0.288
Albumin, g/L	41.2 (37.5–45.4)	41.4 (37.5–45.4)	41.0 (38.5–45.8)	0.632
C-reactive protein, mg/L	2.87 (0.11–7.49)	2.87 (0.12–7.20)	2.12 (0.09–9.71)	0.951
Lymphocyte count, ×10^9^/L	1.85 (1.40–2.35)	1.85 (1.40–2.33)	1.72 (1.50–2.42)	0.889
Platelet count, ×10^9^/L	252 (216–293)	252 (217–284)	254 (212–323)	0.714
Neutrophil count, ×10^9^/L	4.26 (3.11–5.56)	4.16 (2.68–5.44)	5.03 (4.35–6.14)	0.087
Monocyte count, ×10^9^/L	0.54 (0.42–0.65)	0.55 (0.43–0.66)	0.53 (0.39–0.63)	0.352
NLR	2.37 (1.32–3.58)	2.34 (1.30–3.56)	2.61 (1.97–4.28)	0.509
MLR	0.279 (0.196–0.433)	0.282 (0.198–0.463)	0.241 (0.181–0.402)	0.227
PNI	52.0 (46.6–56.3)	51.9 (46.2–56.4)	53.2 (47.2–55.8)	0.542
PLR	135 (106–181)	139 (105–181)	128 (110–182)	0.960
CALLY index	2.85 (0.70–75.39)	2.85 (0.82–61.93)	19.87 (0.66–124.35)	0.708
Soft pancreatic texture	90 (75.0)	78 (75.0)	12 (75.0)	1.000
Pathologic diagnosis/indication for PD				0.025
Pancreatic ductal adenocarcinoma	68 (56.7)	61 (58.7)	7 (43.8)	
Ampullary adenocarcinoma	28 (23.3)	20 (19.2)	8 (50.0)	
Distal cholangiocarcinoma	18 (15.0)	18 (17.3)	0 (0.0)	
Duodenal adenocarcinoma	6 (5.0)	5 (4.8)	1 (6.3)	
Pancreatic duct diameter, mm	3.00 (2.00–3.00)	3.00 (2.00–3.25)	2.00 (1.00–2.00)	<0.001
Operative time, min	360 (300–420)	360 (300–420)	390 (300–428)	0.568
Estimated blood loss, mL	300 (200–500)	300 (200–500)	500 (200–500)	0.103

Data are median (interquartile range) or *n* (%). Continuous variables were compared using the Mann–Whitney U test; binary categorical variables were compared using Fisher’s exact test, and the four-category pathologic diagnosis distribution using the Fisher–Freeman–Halton exact test. CR-POPF, clinically relevant postoperative pancreatic fistula; BMI, body mass index; NLR, neutrophil-to-lymphocyte ratio; MLR, monocyte-to-lymphocyte ratio; PNI, prognostic nutritional index; PLR, platelet-to-lymphocyte ratio; CALLY, C-reactive protein–albumin–lymphocyte index; PD, pancreaticoduodenectomy.

**Table 2:** Repeated cross-validated model performance and calibration.

Model	Predictors	AUC (95% CI)	Average Precision (95% CI)	Brier Score (95% CI)	Brier Skill	Calibration Intercept	Calibration Slope
Integrated random forest	Duct diameter; blood loss; NLR	0.721 (0.576–0.850)	0.294 (0.162–0.542)	0.108 (0.069–0.151)	0.069	−0.092	0.971
Anatomical/intraoperative random forest	Duct diameter; blood loss	0.760 (0.638–0.868)	0.311 (0.176–0.559)	0.103 (0.066–0.144)	0.106	−0.185	0.900
Penalized logistic regression	Duct diameter; blood loss; NLR	0.736 (0.596–0.856)	0.338 (0.179–0.593)	0.107 (0.072–0.146)	0.072	−0.059	1.093

**Table 3:** Incremental-value comparisons.

Comparison	Difference in AUC (95% CI)	Interpretation
Integrated RF vs. anatomical/intraoperative RF	−0.039 (−0.096 to 0.019)	No measurable incremental benefit from NLR
Penalized logistic regression vs. anatomical/intraoperative RF	−0.025 (−0.081 to 0.031)	No clear performance advantage
Integrated RF vs. penalized logistic regression	−0.014 (−0.075 to 0.046)	No clear discrimination advantage for random forest

**Table 4:** Expanded clinicopathological and inflammatory–nutritional random-forest sensitivity analyses.

Model	AUC (95% CI)	Average Precision	Brier Score	ΔAUC vs. Expanded Baseline (95% CI)
Expanded baseline: duct diameter + blood loss + texture + tumor origin	0.743 (0.601–0.862)	0.310	0.106	Reference
Expanded baseline + NLR	0.715 (0.569–0.836)	0.265	0.109	−0.028 (−0.055 to −0.003)
Expanded baseline + MLR	0.779 (0.650–0.888)	0.419	0.102	0.037 (−0.017 to 0.096)
Expanded baseline + PLR	0.703 (0.553–0.834)	0.320	0.108	−0.040 (−0.078 to −0.007)
Expanded baseline + PNI	0.739 (0.597–0.859)	0.341	0.105	−0.004 (−0.046 to 0.039)
Expanded baseline + CALLY	0.710 (0.561–0.838)	0.336	0.108	−0.032 (−0.080 to 0.007)

Expanded baseline predictors were pancreatic duct diameter, estimated blood loss, pancreatic texture, and four-level tumor origin (encoded with pancreatic ductal adenocarcinoma as the reference). Each inflammatory–nutritional marker was added separately. Predictions were patient-level averages of out-of-fold probabilities from 10-times-repeated stratified five-fold cross-validation using the same fixed random-forest specification. AUC confidence intervals and paired ΔAUC intervals were obtained from 3000 patient-level bootstrap resamples. These post hoc analyses were exploratory and were not used to select a final clinical model. The tumor-origin category is interpreted retrospectively; these analyses do not establish that all expanded-model inputs were available before or during surgery.

## Data Availability

De-identified patient-level data are not publicly available because of ethical and institutional confidentiality restrictions. Data may be made available by the corresponding author upon reasonable request and subject to ethical and institutional approvals.

## Supplementary Materials

The following supporting information can be downloaded at https://www.mdpi.com/article/10.3390/jcm15218379/s1. Supplementary Table S1, compares available characteristics between patients included in and excluded from the analytical cohort.

## References

[1] Bassi C., Marchegiani G., Dervenis C., Sarr M., Hilal M.A., Adham M., Allen P., Andersson R., Asbun H.J., Besselink M.G. (2017). The 2016 update of the International Study Group (ISGPS) definition and grading of postoperative pancreatic fistula: 11 Years After. Surgery.

[2] Callery M.P., Pratt W.B., Kent T.S., Chaikof E.L., Vollmer C.M. (2013). A Prospectively Validated Clinical Risk Score Accurately Predicts Pancreatic Fistula after Pancreatoduodenectomy. J. Am. Coll. Surg..

[3] Mungroop T.H., van Rijssen L.B., van Klaveren D., Smits F.J., van Woerden V., Linnemann R.J., de Pastena M., Klompmaker S., Marchegiani G., Ecker B.L. (2019). Alternative Fistula Risk Score for Pancreatoduodenectomy (a-FRS): Design and international external validation. Ann. Surg..

[4] Pande R., Halle-Smith J.M., Phelan L., Thorne T., Panikkar M., Hodson J., Roberts K.J., Arshad A., Connor S., Conlon K.C. (2022). External validation of postoperative pancreatic fistula prediction scores in pancreatoduodenectomy: A systematic review and meta-analysis. HPB.

[5] You J., Fu Y., Cai H., Wang X., Li Y., Zhang M., Tang J., Gao P., Cai Y., Peng B. (2024). Independent external validation and comparison of existing pancreatic fistula risk scores after laparoscopic pancreaticoduodenectomy with Bing’s pancreaticojejunostomy. J. Gastrointest. Surg..

[6] Limbu Y., Acharya B.P., Raut S., Regmee S., Ghimire R., Maharjan D.K., Thapa P.B. (2024). Validation of the Updated Alternate Fistula Risk Score for Prediction of Postoperative Pancreatic Fistula After Pancreatoduodenectomy. JGH Open.

[7] Cheok S.H.X., Lim T.W., Chua D.W., Yeo D.B.X., Tan T.Q.W., Teo J.-Y., Koh Y.-X., Cheow P.-C., Ooi L.L.P.J., Chung A.Y.F. (2026). External validation of the original, alternative, updated alternative, and auditing Fistula Risk Score for the prediction of post-operative pancreatic fistula after minimally invasive and open pancreatoduodenectomy. Surg. Endosc..

[8] Han I.W., Cho K., Ryu Y., Shin S.H., Heo J.S., Choi D.W., Chung M.J., Kwon O.C., Cho B.H. (2020). Risk prediction platform for pancreatic fistula after pancreatoduodenectomy using artificial intelligence. World J. Gastroenterol..

[9] Long Z.-D., Lu C., Xia X.-G., Chen B., Xing Z.-X., Bie L., Zhou P., Ma Z.-L., Wang R. (2022). Personal predictive model based on systemic inflammation markers for estimation of postoperative pancreatic fistula following pancreaticoduodenectomy. World J. Gastrointest. Surg..

[10] Funamizu N., Utsunomiya T., Honjo M., Ito C., Shine M., Uraoka M., Nagaoka T., Tamura K., Sakamoto K., Ogawa K. (2022). Preoperative C-Reactive Protein-to-Albumin Ratio Predicts Postoperative Pancreatic Fistula following Pancreatoduodenectomy: A Single-Center, Retrospective Study. Curr. Oncol..

[11] Utsumi M., Aoki H., Nagahisa S., Nishimura S., Une Y., Kimura Y., Taniguchi F., Arata T., Katsuda K., Tanakaya K. (2020). Preoperative Nutritional Assessment Using the Controlling Nutritional Status Score to Predict Pancreatic Fistula After Pancreaticoduodenectomy. In Vivo.

[12] Ganjouei A.A., Romero-Hernandez F., Wang J.J., Casey M., Frye W., Hoffman D., Hirose K., Nakakura E., Corvera C., Maker A.V. (2023). A Machine Learning Approach to Predict Postoperative Pancreatic Fistula After Pancreaticoduodenectomy Using Only Preoperatively Known Data. Ann. Surg. Oncol..

[13] Verma A.B., Balian J., Hadaya J., Premji A., Shimizu T., Donahue T., Benharash P. (2024). Machine Learning–based Prediction of Postoperative Pancreatic Fistula Following Pancreaticoduodenectomy. Ann. Surg..

[14] Lee W., Park H.J., Lee H.-J., Song K.B., Hwang D.W., Lee J.H., Lim K., Ko Y., Kim H.J., Kim K.W. (2024). Deep learning-based prediction of post-pancreaticoduodenectomy pancreatic fistula. Sci. Rep..

[15] Wang Q., Wang Z., Liu F., Wang Z., Ni Q., Chang H. (2025). Machine learning-based prediction of postoperative pancreatic fistula after laparoscopic pancreaticoduodenectomy. BMC Surg..

[16] Aydin M.C., Ozsay O., Karabulut K., Bircan R., Atalay F., Ors M.B. (2025). Risk Factors For Progression From Biochemical Leak to Clinically Relevant Postoperative Pancreatic Fistula After Pancreaticoduodenectomy—The Key of the Lock: Prognostic Nutritional Index. Turk. J. Gastroenterol..

[17] Garnier J., Alfano M.-S., Robin F., Ewald J., Al Farai A., Palen A., Sebai A., Mokart D., Delpero J.-R., Sulpice L. (2023). Establishment and external validation of neutrophil-to-lymphocyte ratio in excluding postoperative pancreatic fistula after pancreatoduodenectomy. BJS Open.

[18] Marchegiani G., Barreto S.G., Bannone E., Sarr M., Vollmer C.M., Connor S., Falconi M., Besselink M.G., Salvia R., Wolfgang C.L. (2021). Postpancreatectomy Acute Pancreatitis (PPAP): Definition and grading from the International Study Group for Pancreatic Surgery (ISGPS). Ann. Surg..

[19] Iida H., Tani M., Komeda K., Nomi T., Matsushima H., Tanaka S., Ueno M., Nakai T., Maehira H., Mori H. (2022). Superiority of CRP-albumin-lymphocyte index (CALLY index) as a non-invasive prognostic biomarker after hepatectomy for hepatocellular carcinoma. HPB.

[20] Vickers A.J., Elkin E.B. (2006). Decision Curve Analysis: A Novel Method for Evaluating Prediction Models. Med. Decis. Mak..

[21] Collins G.S., Moons K.G.M., Dhiman P., Riley R.D., Beam A.L., Van Calster B., Ghassemi M., Liu X., Reitsma J.B., van Smeden M. (2024). TRIPOD + AI statement: Updated guidance for reporting clinical prediction models that use regression or machine learning methods. BMJ.

