# Supplementary material for: Internal Evaluation of a Machine Learning-Based Random Forest Model for Clinically Relevant Postoperative Pancreatic Fistula After Pancreaticoduodenectomy: Limited Incremental Value of Inflammatory–Nutritional Markers
Source: J Clin Med. 2026 Oct 28;15(21):8379. doi: 10.3390/jcm15218379 (PMC13763827; doi:10.3390/jcm15218379)
Supplement: Supplementary file 1 [file jcm-15-08379-s001.zip › jcm-4492740-supplementary.pdf]

Supplementary Table S1. Comparison of available characteristics between patients included in and excluded from the analytical cohort

Variable	Included cohort (n=120)	Excluded cohort (n=38)	p value
Age, years	69.0 (63.8–76.0)	66.0 (60.5–72.8)	0.266
Male sex	79 (65.8)	23 (60.5)	0.564
Diabetes mellitus	67 (55.8)	24 (63.2)	0.457
Hemoglobin, g/dL	13.4 (11.7–14.7)	12.2 (11.3–14.2)	0.116
Total bilirubin, mg/dL	0.98 (0.61–3.09)	1.00 (0.71–2.01)	0.717
Albumin, g/L	41.2 (37.5–45.4)	41.0 (36.0–44.8)	0.566
C-reactive protein, mg/L	2.87 (0.11–7.49)	2.72 (0.13–7.40)	0.864
Lymphocyte count, $\times 10^9$/L	1.85 (1.40–2.35)	1.84 (1.41–2.21)	0.842
Platelet count, $\times 10^9$/L	252 (216–293)	248 (209–282)	0.725
Neutrophil count, $\times 10^9$/L	4.26 (3.11–5.56)	4.26 (3.07–5.50)	0.893
NLR	2.37 (1.32–3.58)	2.30 (1.50–3.04)	0.956
PNI	52.0 (46.6–56.3)	51.4 (45.6–54.4)	0.547
PLR	135 (106–181)	139 (106–180)	0.987
CALLY index	2.85 (0.70–75.39)	3.33 (0.98–67.16)	0.799
Soft pancreatic texture	90 (75.0)	27 (71.1)	0.673
Operative time, min	360 (300–420)	330 (300–420)	0.510
Estimated blood loss, mL	300 (200–500)	500 (200–500)	0.209

Data are median (interquartile range) or n (%). Continuous variables were compared using the Mann–Whitney U test and categorical variables using Fisher's exact test. Pancreatic duct diameter is not presented because absence of its documentation defined the excluded cohort. NLR, neutrophil-to-lymphocyte ratio; PNI, prognostic nutritional index; PLR, platelet-to-lymphocyte ratio; CALLY, C-reactive protein–albumin–lymphocyte index.
